# Clinical usefulness of image‐enhanced endoscopy for the diagnosis of ulcerative colitis‐associated neoplasia

**DOI:** 10.1002/deo2.325

**Published:** 2024-01-06

**Authors:** Kaoru Takabayashi, Motohiko Kato, Takanori Kanai

**Affiliations:** ^1^ Center for Diagnostic and Therapeutic Endoscopy, Keio University School of Medicine Tokyo Japan; ^2^ Department of Internal Medicine Division of Gastroenterology and Hepatology Keio University School of Medicine Tokyo Japan

**Keywords:** dye‐chromoendoscopy, linked‐color imaging, narrow band imaging, texture and color enhancement imaging, ulcerative colitis‐associated neoplasia

## Abstract

Patients with a long history of ulcerative colitis (UC) are at risk of developing a significant complication known as UC‐associated neoplasia (UCAN). To reduce the risk of UCAN and the associated mortality, the current guidelines recommend initiating surveillance colonoscopy 8–10 years after confirmation of UC diagnosis. In recent years, advancements in endoscopic diagnostic technologies, including magnifying and image‐enhancing techniques, have allowed for the production of high‐contrast images that emphasize mucosal structures, vascular patterns, and color tones. Recently, image‐enhanced endoscopy technologies have become available and offer the potential to improve the qualitative endoscopic assessment of UCAN. The use of high‐definition chromoendoscopy enables the evaluation of subtle mucosal patterns in the colon. Magnifying narrow‐band imaging facilitates the visualization of mucosal vascular structures. Texture and color enhancement imaging processes structure, color tone, and brightness aspects more appropriately, whereas linked color imaging optimizes the emphasis on mucosal and vascular redness. Both techniques are expected to excel in the depiction of subtle color variations and mucosal changes characteristic of UCAN. This article provides an overview of the current status and future challenges regarding the use of various image‐enhanced endoscopy techniques in the diagnosis of UCAN.

## INTRODUCTION

Ulcerative colitis (UC) is a chronic, persistent inflammatory bowel disease of unknown etiology characterized by recurrent episodes of relapse and remission.^1^ Typically, it begins with mild inflammation of the rectum and progresses to extensive inflammation throughout the colon. Persistent inflammation increases the risk of UC‐associated neoplasia (UCAN) development and the potential need for future total colectomy. The cumulative incidence of UCAN associated with long‐term UC has been reported to reach approximately 20% after 30 years,^2^ although the reported rates may vary. To reduce the risk of UCAN and associated mortality, the current guidelines recommend initiating surveillance colonoscopy 8–10 years after the confirmation of UC diagnosis.^3^, ^4^, ^5^

**FIGURE 1:**
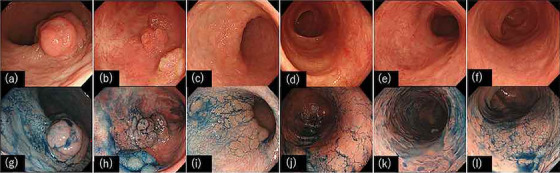
Representative endoscopic images of ulcerative colitis‐associated dysplasia according to the SCENIC consensus statement. (a–f) White light Images, (g–l) chromoendoscopic images, (a, g) pedunculated, (b, h) sessile, (c, i) superficial elevated, (d, j) flat, (e, k) depressed, and (f, l) invisible dysplasia.

Despite rapid advancements in endoscopic equipment and diagnostic technologies in recent years, the early detection of UCAN remains challenging owing to the complex background of inflamed mucosa in UC and the morphological diversity of lesions. The Surveillance for Colorectal Endoscopic Neoplasia Detection and Management in Inflammatory Bowel Disease Patients: International Consensus Recommendations (SCENIC) statement ^5^, ^6^ and the American Gastroenterological Association (AGA) guidelines^7^ classify UCAN into five types: pedunculated, sessile, superficially elevated, flat, and depressed lesions (Figure 1 and Table 1). Previous reports based on these criteria have indicated that 64% of high‐grade dysplasia lesions are elevated, while 30% are flat‐type.^8^ The detection of elevated lesions is considered possible with white‐light imaging (WLI); however, some lesions classified as flat, including so‐called “invisible dysplasia,” may be challenging to detect with WLI. The identification of flat lesions, including those in these cases, poses a significant challenge for an early UCAN diagnosis.

**TABLE 1:** Endoscopic morphologic features of ulcerative colitis‐associated dysplasia.

**Visible dysplasia**	Dysplasia identified on targeted biopsies from a lesion visualized at colonoscopy
Polypoid lesion	Lesion protruding from the mucosa into the lumen ≥2.5 mm
Pedunculated	Lesion attached to the mucosa by a stalk
Sessile	Lesion not attached to the mucosa by a stalk entire base is contiguous with the mucosa
Nonpolypoid lesion	Lesion with little (<2.5 mm) or no protrusion above the mucosa
Superficial elevated	Lesion with protrusion but <2.5 mm above the lumen (less than the height of a closed cup of a biopsy forceps)
Flat	Lesion without protrusion above the mucosa
Depressed	Lesion with at least a portion depressed below the level of the mucosa
**Invisible dysplasia**	Dysplasia identified on random (non‐targeted) biopsies of colon mucosa without a visible lesion

The SCENIC consensus statement recommends the use of image‐enhanced endoscopy (IEE) for UCAN diagnosis. However, specific guidelines on which IEE is used for particular cases or which IEE methods are useful for detection or delineation are not explicitly stated. Therefore, this section provides an overview of how IEE, developed to date, is utilized in the diagnosis of UCAN, focusing on the latest findings.

## DYE CHROMOENDOSCOPY

The 2015 SCENIC consensus statement recommended chromoendoscopy combined with dye spraying for UCAN detection (Figure 1). This statement proposes a method involving the application of a diluted indigo carmine solution throughout the colon. In cases where mucosal surfaces or lesions suggestive of UCAN were identified, a normal concentration of indigo carmine solution was sprayed for detailed examination. This technique is considered beneficial because the application of indigo carmine or methylene blue enhances the visibility of fine structures on the mucosal surface. A meta‐analysis evaluating the effectiveness of this method reported that the detection rate of UCAN nearly doubled when chromoendoscopy was combined with dye spraying. This result led to the recommendation of chromoendoscopy with dye spraying.^9^, ^10^, ^11^ Subsequent studies, such as that by Carballal et al.,^12^ demonstrated that even non‐experts achieved UCAN detection rates equivalent to those of experts through chromoendoscopy, suggesting the effectiveness of chromoendoscopy for UCAN surveillance, irrespective of their experience or skill. Watanabe et al.^13^ compared the UCAN detection rates between chromoendoscopy‐assisted targeted biopsies and random biopsies under conventional observation. They reported the noninferiority of targeted biopsies to random biopsies, further supporting the utility of chromoendoscopy for UCAN detection.

**FIGURE 2:**
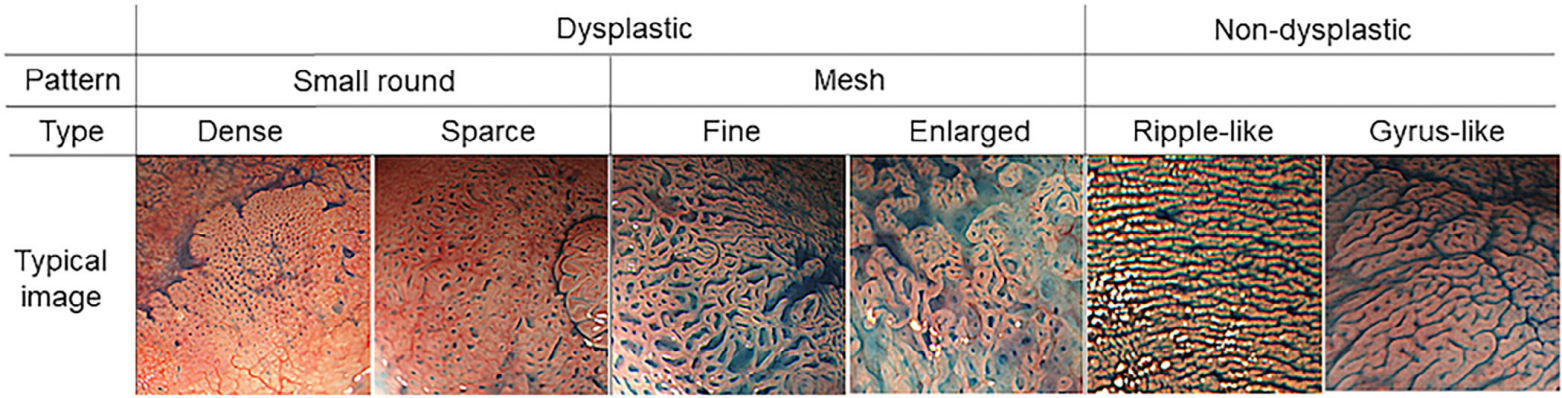
Typical chromoendoscopic mucosal patterns of flat dysplasia. The dysplastic mucosal patterns were classified into two types: small round and mesh‐type patterns. The small round pattern was further subdivided into dense and sparse patterns. The mesh patterns were subdivided into fine and enlarged types. Non‐dysplastic mucosal patterns are divided into two major types: ripple‐like and gyrus‐like.

However, the challenges associated with this method include a significant extension of the examination time and a lack of specific endoscopic findings, clearly indicating the suspicion of UCAN when observing lesions or mucosal changes. These issues need to be addressed in future studies. Amidst these challenges, Takabayashi et al.^14^ reported the features of indigo carmine dye spraying images for flat‐type dysplasia, including invisible dysplasia, which is considered difficult to detect. They retrospectively analyzed the indigo carmine dye‐sprayed endoscopic images of 63 lesions with flat dysplasia and classified the images into two main patterns: small round and mesh patterns (Figure 2). They reported that approximately 70% of lesions with a small round pattern exhibited high histopathological malignancy, ranging from high‐grade dysplasia to carcinoma. Among lesions with a mesh pattern, approximately half were histopathologically low‐grade dysplasia. However, the sensitivity and specificity of each finding have not been studied, and further investigation is required to assess the usefulness of these observations.

## NARROW‐BAND IMAGING

Narrow‐band imaging (NBI) is an optical endoscopic technology that allows clear visualization of the superficial microvascular structures of the gastrointestinal mucosa.^15^ NBI utilizes narrowband illumination created by two NBI filters: blue light at 415 ± 30 nm and green light at 540 ± 30 nm. The 415 nm light corresponded to the absorption band of hemoglobin. NBI combined with magnifying endoscopy is commonly used to identify early cancers of the oropharynx, esophagus, stomach, and colon. Particularly, for colorectal lesions, the pit pattern classification proposed by Kudo et al.^16^ and the Japan NBI Expert Team (JNET) classification proposed by Sano et al.^17^ were employed to differentiate between tumors and non‐tumors in patients without UC.

**FIGURE 3:**
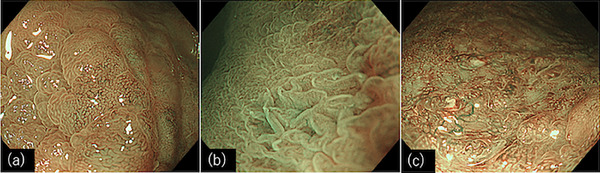
Representative endoscopic images of magnifying narrow‐band imaging as proposed by Matsumoto et al. The surface structure was classified as (a) honeycomb‐like, (b) villous, and (c) tortuous patterns.

**FIGURE 4:**
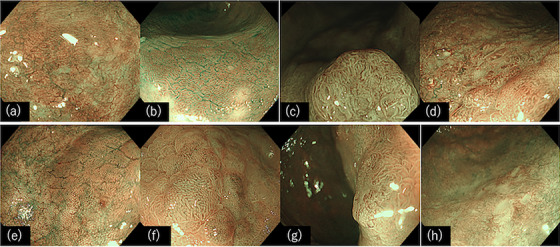
Representative endoscopic images of magnifying narrow‐band imaging as proposed by Nishiyama et al. The surface patterns are classified as (a) unclear, (b) regular, (c) irregular, and/or (d) amorphous. The vascular patterns are classified as: (e) like that of the background mucosa, (f) regular, (c) irregular, or (d) avascular.

The application of NBI for UCAN diagnosis was first reported by East and colleagues.^18^ They reported that the diagnosis of UCAN using only Kudo's pit pattern classification, which is typically used to diagnose sporadic neoplasms, was challenging. Applying only the conventional Kudo's pit pattern classification may lead to discrepancies between the expected and actual tissue images. Therefore, they concluded that a diagnosis based not only on the pit pattern but also on features observed with NBI, such as the vascular pattern within the tumor, is necessary. They emphasized the importance of proposing new diagnostic criteria based on these considerations. Subsequent studies were conducted to diagnose UCAN using NBI. Matsumoto et al.^19^ visually classified the surface patterns of lesions observed using magnified NBI into honeycomb‐like, villous, and tortuous patterns (Figure 3). They reported that a tortuous pattern suggested the possibility of UCAN. However, Nishiyama et al.^20^ proposed that an irregular/amorphous surface pattern and an irregular/avascular vessel pattern indicate tumorous lesions. They reported that by combining these surface and vessel patterns, tumorous lesions could be accurately differentiated in 91% of the cases (Figure 4). Despite these reports, standardized diagnostic criteria for UCAN based on NBI have not yet been established. This is because these studies mainly focused on elevated or superficially elevated types of UCAN, and there are still several unresolved issues, such as distinguishing between microvessels modified by inflammation and pit structures in conventional sporadic neoplasms, based on NBI findings.

## TEXTURE AND COLOR ENHANCEMENT IMAGING

Texture and color enhancement imaging (TXI) is a novel IEE technology available in the EVIS X1 series developed by the Olympus Corporation. It utilizes a digital IEE method to optimize and synthesize images obtained with WLI in three elements: “structure (texture),” “color tone,” and “brightness”.^21^ In the TXI, the WLI image is first divided into a texture image containing information about surface irregularities and a base image containing brightness components. The two images were individually processed (enhanced) and combined to produce a composite output (TXI Mode2). Color tone enhancement processing was applied to the TXI Mode2 image to generate the TXI Mode1 image. By separating these three elements, the structure, color tone, and brightness can be emphasized more appropriately. TXI Mode2 emphasizes the structure and brightness with color tones similar to those of WLI, reducing the examination stress associated with IEE. The standard TXI Mode1 includes color tone enhancement, leading to improved observability of lesions with subtle color changes.

**FIGURE 5:**
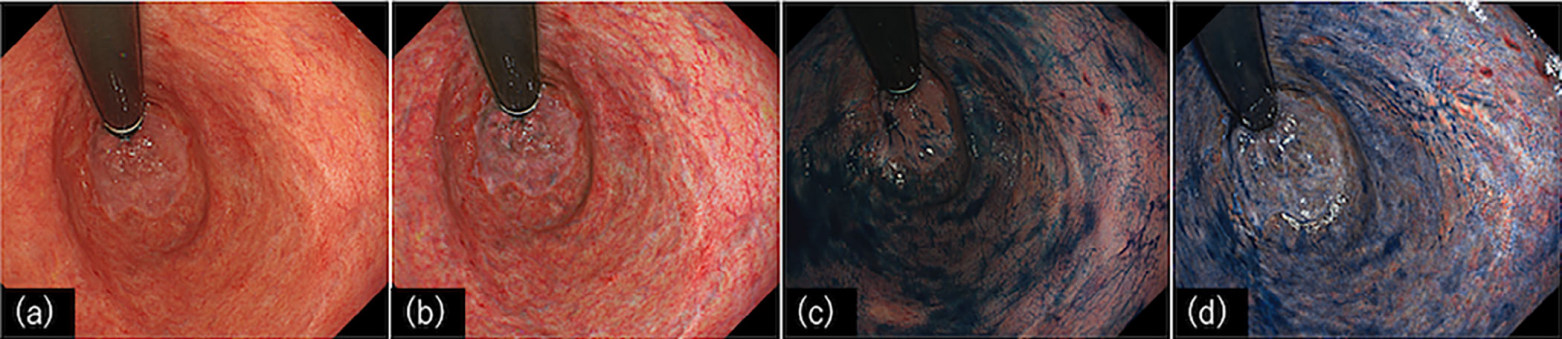
Representative endoscopic images of texture and color enhancement imaging. (a) White light imaging; (b) texture and color enhancement imaging mode1, (c) white light imaging with indigo carmine dye spray; (d) texture and color enhancement imaging mode1 with indigo carmine dye spray.

The anticipated utility of TXI based on their features includes: (1) improved visibility of superficial digestive tract tumors and (2) enhanced visibility of non‐tumorous lesions and biological structures. However, because its clinical use is relatively recent, there has been only one reported case of its utility for UCAN. Takabayashi et al.^22^ reported cases in which the use of TXI Mode1 improved the clarity of lesion boundaries in UCAN, which were challenging to recognize with WLI, TXI, and chromoendoscopy with indigo carmine dye spraying alone (Figure 5). The slight irregularities on the mucosal surface caused by indigo carmine spraying and the color contrast between the mucosal surface and the surrounding normal mucosa were both emphasized by TXI, resulting in a clearer boundary with the normal mucosa. Although further investigation through case accumulation is necessary, the combination of chromoendoscopy and TXI is expected to enhance the UCAN detection rate.

## LINKED‐COLOR IMAGING

Linked‐color imaging (LCI) is a novel IEE available in the LASEREO endoscopic system developed by the Fujifilm Corporation in Tokyo, Japan. LCI provides an extended color range obtained through narrowband illumination pre‐processing and color separation post‐processing. This allows for better recognition of slight differences in colors by reallocating blue, green, and red to enhance color discrimination. Particularly in LCI, it becomes possible to display images with enhanced contrast by overlaying violet light at 410 nm, making red‐dominant elements, such as mucosa and vessels appear redder, and white‐dominant elements, such as ulcers and scars appear whiter. Therefore, LCI is expected to improve the visualization of inflammatory changes, such as those resulting from inflammation or atrophy, compared with conventional WLI.

Similar to TXI, there are only case reports on the utility of LCI for the diagnosis of UCAN. Hisamatsu et al.^23^ reported cases in which areas initially recognized as indistinct red mucosa under WLI became clearly demarcated as red regions when observed with LCI, leading to a diagnosis of flat‐type UCAN. Kanmura et al.^24^ also focused on red mucosa with clarified boundaries using LCI, where the addition of indigo carmine spray further emphasized the boundaries of the red mucosa and made the irregularities of lesions clearer.

**FIGURE 6:**
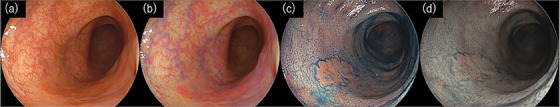
Representative endoscopic images of linked‐color imaging. (a) White light imaging, (b) linked‐color imaging, (c) white light imaging with indigo carmine dye spraying, and (d) linked‐color imaging (LCI) with indigo carmine dye spraying.

Similar to TXI, further investigations using case studies are necessary. However, the potential utility of LCI in detecting UCAN in the red mucosa after inflammation is expected because of its ability to enhance the contrast between red and white elements (Figure 6).

## DISCUSSION

Diagnosing regular colorectal cancer that does not originate in the inflamed mucosa is generally not as challenging given the application of imaging‐enhanced observations, such as pit pattern diagnosis and NBI during endoscopy. As long as it comes into the endoscope's field of view, not only is the presence able to be observed, but the diagnosis of the extent and depth is also highly accurate through image‐enhanced observations, such as chromoendoscopy, NBI, and magnifying observation. Contrastingly, diagnosis of the presence and extent of UCAN is not straightforward. This is because the background mucosa often presents diverse images due to inflammation, and UCAN poses several difficulties because it exhibits various macroscopic forms and pit patterns. Furthermore, it is often challenging to diagnose the depth, as mucosal lesions may persist while progressing invasively to advanced cancers, and the surface characteristics do not necessarily reflect those of deeper layers. However, even in challenging situations, conducting surveillance colonoscopies for UCAN is important. Although there are no randomized controlled trials addressing the reduction of colorectal cancer incidence or mortality through surveillance colonoscopy, multiple cohort, and case‐control studies have demonstrated that surveillance colonoscopy can lead to the early detection of colorectal cancer, contributing to favorable outcomes and proving cost‐effectiveness. In a retrospective cohort study of patients with inflammatory bowel disease, Lutgens et al. reported that the proportion of stage 0 and I in the TNM classification at the time of colorectal cancer diagnosis was 52% in the surveillance colonoscopy group and 24% in the non‐surveillance group (*p* = 0.004). The 5‐year survival rate after colorectal cancer diagnosis was 100% in the surveillance colonoscopy group compared to 74% in the non‐surveillance group (*p* = 0.042).^25^ This suggests that more tumors were detected early in the surveillance group, leading to significantly fewer deaths from colorectal cancer in the surveillance group. Furthermore, Hata et al. reported that patients undergoing regular surveillance colonoscopy had better overall survival than those who did not undergo surveillance.^26^ This underscores the importance of endoscopic assessment of inflammatory activity and early detection of UCAN through surveillance colonoscopy in the management of patients with UC.

Given this background, there has been extensive discussion on the optimal endoscopic observation methods for surveillance. Regarding the endoscopic observation methods useful for UCAN detection, it has been reported in a meta‐analysis that the tumor detection rate per patient is higher with chromoendoscopy using indigo carmine or methylene blue compared to conventional white‐light observation (relative risk [RR] 1.50; 95% confidence interval [CI], 1.08–2.10).^27^ However, the usefulness of chromoendoscopy in the detection of UCAN during surveillance is not observed when compared to high‐definition white‐light observation (RR 1.36; 95% CI, 0.84–2.18). Notably, there is no significant difference in the comparison between chromoendoscopy and NBI, and in the comparison at the tumor unit level, NBI observation is reported to be inferior to high‐definition white‐light observation (RR 0.62; 95% CI, 0.44–0.88) according to a meta‐analysis.^28^, ^29^, ^30^ Furthermore, the utility of UCAN detection using techniques, such as TXI or LCI has not been thoroughly investigated, and while not discussed in detail here, the reports to date have not confirmed the effectiveness of autofluorescence imaging (AFI) in UCAN detection.^31^, ^32^ Endomicroscopy, while suggested to be useful in the detailed examination of lesions with a confirmed diagnosis of UCAN, has been reported to be less effective in detection.^33^, ^34^, ^35^, ^36^ In 2019, Iacucci et al.^37^ proposed an endoscopic classification system to distinguish UCAN and sporadic neoplasia by combining various endoscopic findings. Although this classification, which comprehensively assesses lesion morphology, color tone, margin, surface structure, and vascular architecture to diagnose UCAN, recommends combining four observations: non‐polypoid lesion, irregular vessel architecture, irregular surface pattern, and ulceration within the lesion, with an overall diagnostic accuracy of 71% (sensitivity: 65%, specificity: 81%, positive predictive value: 86%, and negative predictive value: 57%). Thus, it is important to recognize the current limitations of using IEE for the endoscopic differentiation of UCAN from sporadic neoplasia.

Numerous studies utilizing IEE have been reported to date; however, a definitive diagnosis of UCAN has not yet been established. In a recent report by Choi et al.,^38^ the cumulative incidence rates were 0.07%, 2.9% at 20 years, and 6.7% at 10, 20, and 30 years old, respectively. Similarly, a study by Kishikawa et al.^39^ reported the rates of 0.7%, 3.2%, and 5.2%, respectively. Although the risk of developing UCAN was not as high as previously reported, it remains an important issue during the long‐term course of UC. Unlike sporadic colorectal cancer, UCAN tends to occur at a younger age, with a higher proportion of poorly differentiated or mucinous cancers. Additionally, lesions that are difficult to recognize endoscopically tend to become more prevalent. Therefore, challenges persist in the diagnosis of UCAN.

**FIGURE 7:**
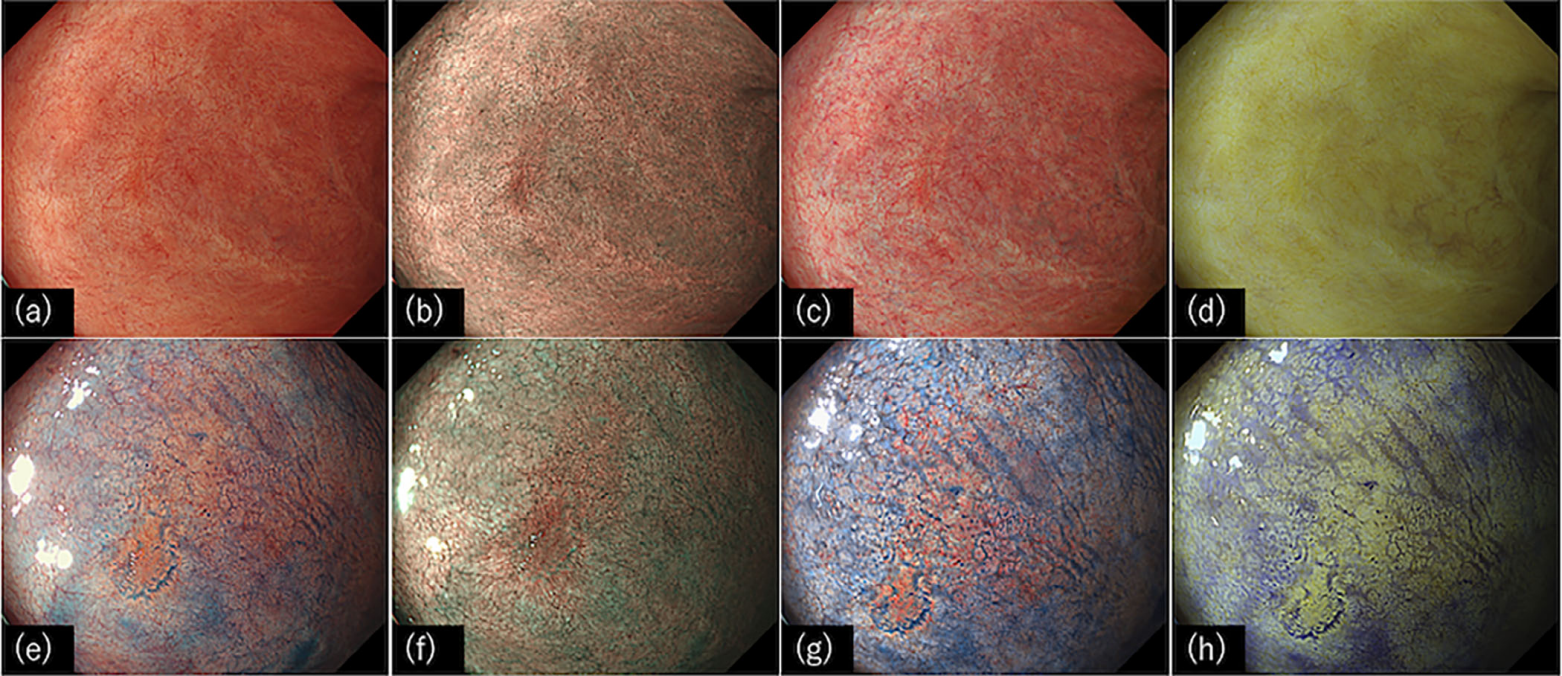
Representative endoscopic images of flat‐type dysplasia with ulcerative colitis using several image‐enhanced endoscopies. (a) White light imaging, (b) narrow‐band imaging, (c) texture and color enhancement Imaging mode1, (d) red dichromatic imaging, (e) white light imaging with indigo carmine dye spraying, (f) magnifying narrow‐band imaging with indigo carmine dye spraying, (g) texture and color enhancement imaging mode1 with indigo carmine dye spraying, and (h) red dichromatic imaging with indigo carmine dye spraying.

In conclusion, this discussion presents insights into the latest understanding of UCAN based on the characteristics of various IEE methods. However, in the future, it will be crucial to conduct studies aimed at establishing a diagnostic approach for UCAN by combining existing and novel IEE techniques, considering their respective characteristics (Figure 7).

## CONFLICT OF INTEREST STATEMENT

None.
